# Suzetrigine: A Selective Inhibitor of the NaV1.8 Sodium Channel

**DOI:** 10.7759/cureus.115406

**Published:** 2026-08-29

**Authors:** Daniel Bach, Kevin Tran, George Wilkins, Zachary Yamada, Paramveer Brar, Sudhakar Pemminati

**Affiliations:** 1 Department of Biomedical Education, California Health Sciences University College of Osteopathic Medicine, Clovis, USA

**Keywords:** nav1.8, nav1.8 inhibitor, nav1.8 sodium channel blocker, perioperative pain management, suzetrigine

## Abstract

Opioid related adverse events are an important perioperative concern despite widespread adoption of combined analgesic approaches for pain management. Suzetrigine, a selective inhibitor of the NaV1.8 sodium channel for the treatment of moderate-to-severe acute pain, enables the use of a novel analgesic class in clinical practice. The goal of this scoping review is to analyze suzetrigine’s pharmacokinetic, pharmacodynamic, and clinical evidence. Utilizing the Preferred Reporting Items for Systematic Reviews and Meta-Analyses extension for Scoping Reviews guidelines, the online databases of PubMed, Embase, and ClinicalTrials.gov (January 2020 to February 2026) were systematically searched for various studies investigating NaV1.8-selective compounds in acute pain contexts. A total of five reviewers screened and extracted data with cross-examination. A total of 10 articles were identified to meet the specified inclusion criteria for this scoping review. These articles were recognized as peer-reviewed publications, clinical trial registry results, regulatory documents, conference abstracts, and preprints. Suzetrigine was found to be most effective when given orally twice daily, and its selectivity for NaV1.8 was up to 30,000 times greater than that of other sodium channel medications pursuing the same effect. Suzetrigine was associated with a decrease in gastrointestinal symptoms when compared to opioid counterparts, a decrease of up to 40%. Mechanistic studies reveal that NaV1.8 inhibition reduces, but does not abolish, pain receptor firing, explaining the meaningful yet incomplete analgesia observed clinically. Suzetrigine is also metabolized by cytochrome P4503A (CYP3A) enzymes, suggesting potential drug interactions. Suzetrigine provides a unique mechanism to relieve acute pain without opioids. Current evidence gaps include limited data across comprehensive surgical populations, multimodal comparative effectiveness of trials, and undefined safety outcomes. Perioperative analgesic regimens can be optimized by prioritizing research addressing areas with insufficient evidence.

## Introduction and background

Surgical procedures cause necessary tissue damage, leading to inevitable activation of the nociceptive pathway and requiring pharmacological intervention in the form of pain control. Opioid receptor agonists have been the cornerstone of moderate-to-severe surgical pain management, but their utility is limited by well-known issues, including respiratory compromise, gastrointestinal slowing, cognitive issues, and the potential for the creation of addictive patterns [1,2]. As we reel from these limitations as a medical community, a new search has been catalyzed: one for an analgesic that provides consolidation of multiple roles similar to what made opioids such a prime option without the significant adverse effects that accompany them [3,4]. Despite considerable progress in multimodal approaches, meaningful proportions of surgical patients continue to experience inadequately controlled pain, underscoring the persistent demand for novel non-opioid therapeutic options [5]. An ideal candidate would provide reliable analgesia while avoiding central nervous system (CNS) depression, organ toxicities, and dependence liability that complicate current pharmacotherapy. Nearly 20% of patients treated with opioids experience an opioid-related adverse drug event, most commonly gastrointestinal effects, CNS effects, pruritus, and urinary retention [6]. Systematic reviews convey that opioid prescribing increases the chance of vomiting more than fourfold compared with opioid-free analgesia, with additional increases in nausea, constipation, dizziness, and drowsiness [7]. These adverse effects impair postoperative recovery, delay mobilization, prolong hospital stays, and consistently raise healthcare costs [6,8].

The initiation and maintenance of electrical signals in the nervous system are mediated by voltage-gated sodium channels. While there are nine mammalian subsets, NaV1.1-1.9, NaV1.7, NaV1.8, and NaV1.9 are preferentially geared toward the sensory components of the peripheral nervous system [9-11]. Each represents a different function, with NaV1.7 handling action potential initiation, NaV1.8 handling repetitive firing during periods of sustained stimulation, and NaV1.9 modulating resting membrane potentials and subthreshold excitability [9,12]. Due to NaV1.8’s unique role, it is particularly attractive as a target for the development of analgesics. Its expression is primarily confined to dorsal root and trigeminal ganglion neurons, with near-zero incidence in cardiac, skeletal muscle, or CNS structures [9,11,13]. This selective expression theoretically allows modulation of pain without the cardiac arrhythmias, cardiac depression, or peripheral weakness otherwise seen with nonselective sodium-channel blockers. Moreover, NaV1.8 demonstrates tetrodotoxin resistance and contributes significantly to nociceptor excitability in the backdrop of inflammation, positioning it as a pertinent target for pain related to tissue injury, especially during the postoperative period [12,13]. As illustrated in Figure 1, open NaV1.8 channels permit sodium influx that supports repetitive action potential firing in peripheral nociceptors, whereas suzetrigine stabilizes the channel in a closed, nonconducting state, reducing sodium entry and attenuating peripheral pain signal transmission.

**Figure 1:**
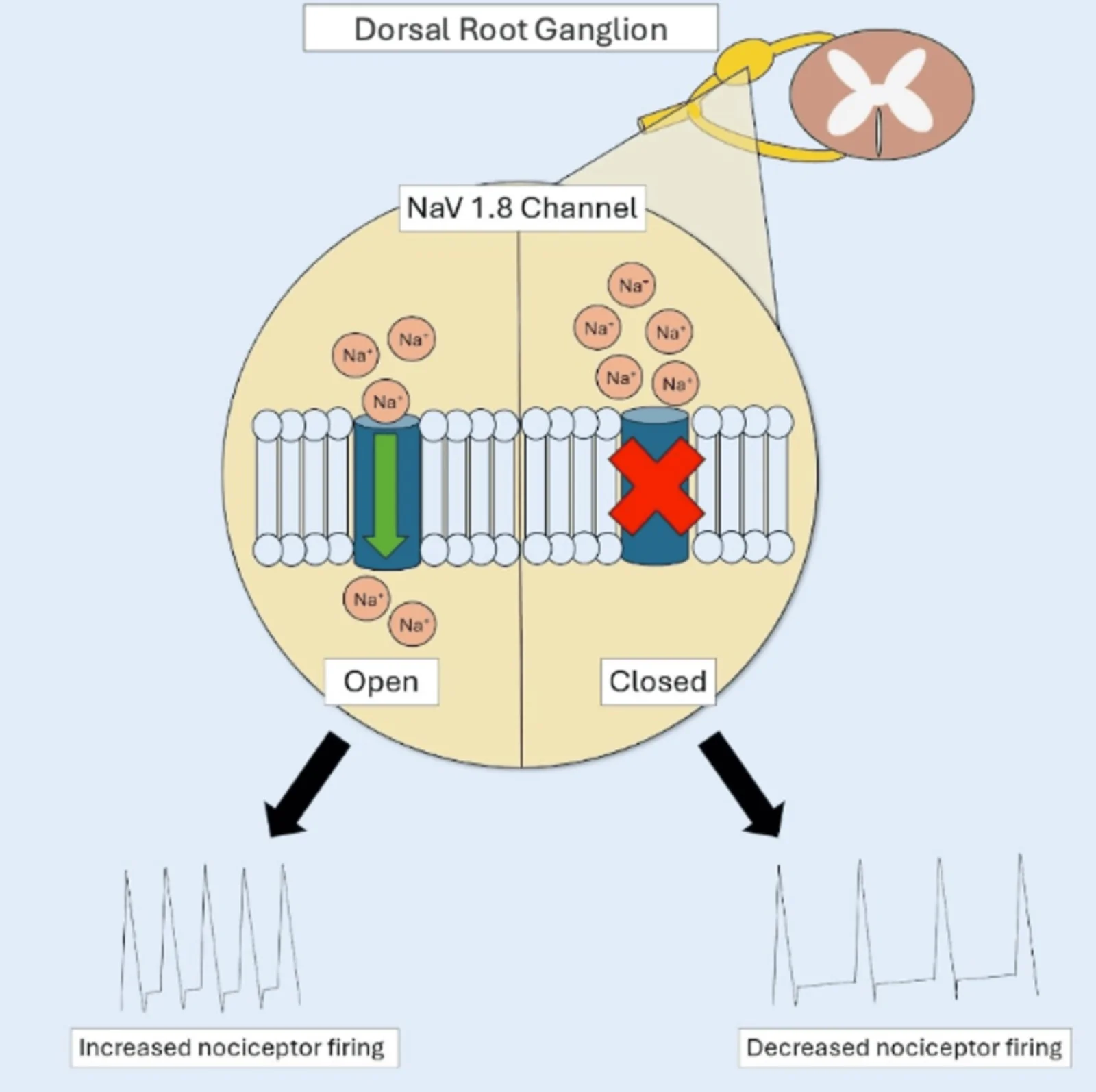
Conceptual illustration of NaV1.8 channel activity in peripheral nociceptors of the dorsal root ganglion and the proposed mechanism of action of suzetrigine Image credits: Kevin Tran; created using PowerPoint (Microsoft Corp., Redmond, WA, USA) tools.

Building upon this, pharmaceutical development has shown exceptional selectivity for NaV1.8. Suzetrigine, formerly designated VX-548, emerged from these clinical trials as the most likely candidate and received United States Food and Drug Administration (FDA) approval in January 2025 under the trade name JOURNAVX [11,14]. The perioperative environment offers an ideal testing ground for evaluating such agents because surgical pain follows predictable temporal trajectories, medication administration can be standardized, and outcomes spanning pain intensity, rescue analgesic consumption, adverse events, and functional recovery can be assessed over clinically meaningful intervals [14,15]. Early randomized investigations have demonstrated that suzetrigine provides measurable analgesia exceeding placebo in established postoperative pain models, with favorable tolerability compared with opioid-containing regimens [16].

Although preliminary data suggest therapeutic promise, evidence supporting NaV1.8-targeted analgesia remains unsynthesized from pharmacodynamic and therapeutic standpoints. This study reviews the medication’s mechanism of action and its effects on postoperative pain control, where opioid medications remain the cornerstone of treatment. A scoping review provides an appropriate framework for addressing this research gap by enabling a comprehensive understanding of the available evidence, clarifying key concepts, and identifying gaps in the evidence [17,18]. This scoping review therefore pursues three objectives: first, to synthesize pharmacokinetic and pharmacodynamic evidence characterizing suzetrigine and related NaV1.8-selective compounds; second, to combine clinical findings regarding analgesic effect, tolerability, and use in acute pain settings; and third, to identify gaps in the evidence that may guide future investigation and advance perioperative implementation. This study was presented at the Annual Research Day in May 2026 at California Health Sciences University College of Osteopathic Medicine, Clovis, California.

## Review

Methods 

This scoping review followed contemporary evidence-mapping standards and the Preferred Reporting Items for Systematic Reviews and Meta-Analyses extension for Scoping Reviews (PRISMA-ScR) [17]. The design emphasized descriptive synthesis rather than quantitative pooling or formal quality appraisal and was informed by the framework of Arksey and O’Malley and subsequent methodological refinements [18,19]. Review procedures were specified in advance in a study protocol before literature identification, screening, and data extraction. The protocol was not registered in a public repository before initiation, which is acknowledged as a limitation.

PubMed, Embase, and ClinicalTrials.gov were searched for records published between January 2020 and February 2026, reflecting the recent clinical development of NaV1.8-selective compounds. The last search date was March 1, 2026. Search strategies combined controlled vocabulary and keywords for voltage-gated sodium channels and specific isoforms, suzetrigine or VX-548, postoperative and acute pain, and pharmacological or clinical outcomes. The study selection process is summarized in Figure 2.

**Figure 2:**
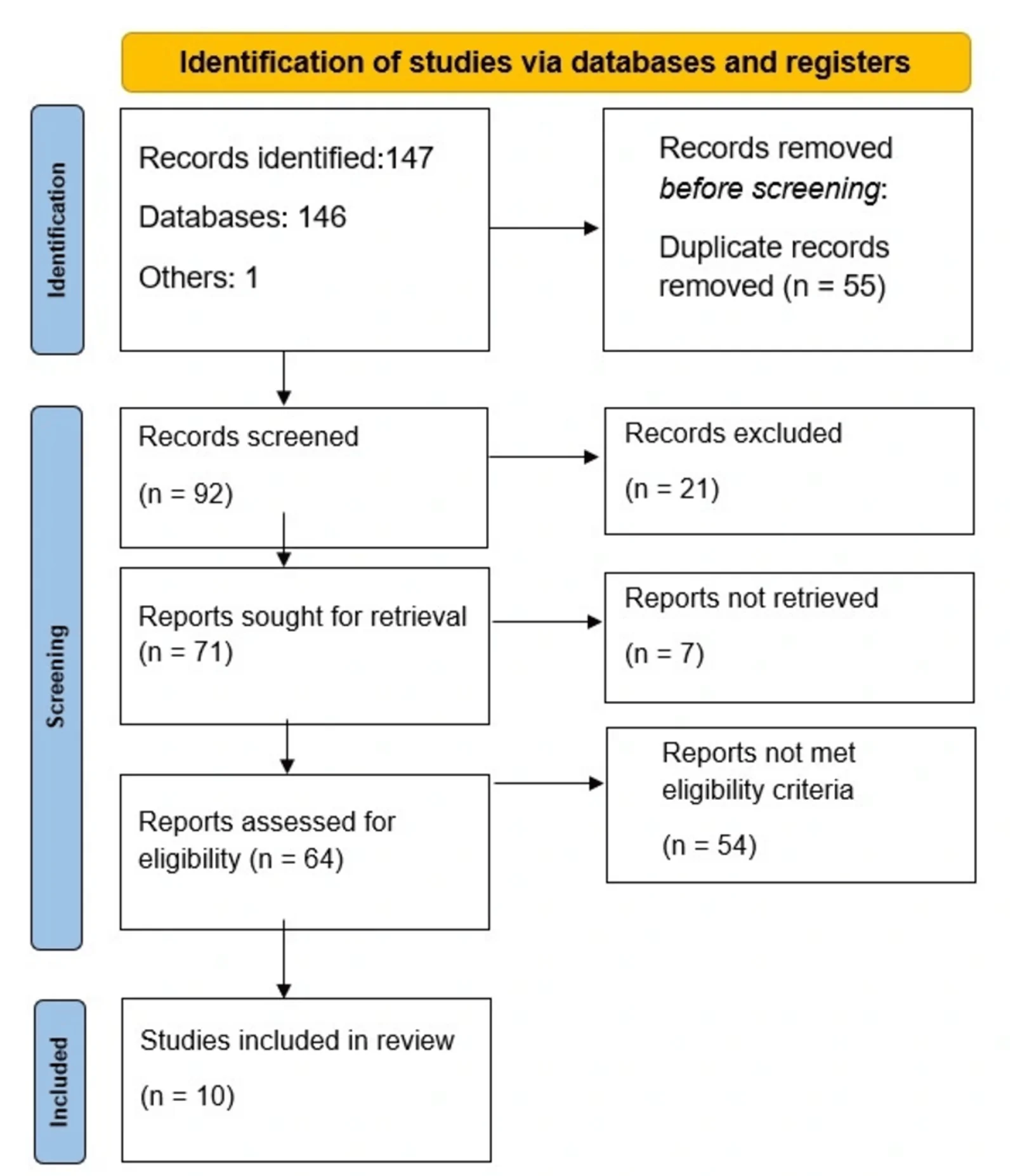
Study selection process

Five reviewers conducted study selection in pairs, with a fifth reviewer available to resolve disagreements. Titles and abstracts were screened first, followed by independent full-text assessment by two reviewers. Discrepancies were resolved through group discussion. Standardized charting captured study design, population or experimental model, pain context, investigational agent and dose, comparator, pharmacokinetic and pharmacodynamic parameters, efficacy outcomes, safety outcomes, and the research questions addressed. At least two reviewers verified the extracted data, with a third resolving any remaining discrepancies. Findings were synthesized narratively across pharmacokinetic, pharmacodynamic, and clinical domains, and each study was mapped to the research question it addressed to maintain transparent reporting consistent with PRISMA-ScR guidance [18]. No meta-analysis or formal risk-of-bias assessment was performed, consistent with scoping-review methodology.

Inclusion Criteria 

Eligible sources examined NaV1.8-selective compounds in adults or systems modeling surgical or incisional pain. Peer-reviewed mechanistic studies, pharmacokinetic and pharmacodynamic investigations, registered clinical trials with publicly available results, regulatory documents, and conference abstracts containing original data were considered. Human mechanistic or pharmacokinetic studies were included when they provided clinically relevant information on target engagement, selectivity, or exposure-response relationships. The clinical scope included perioperative and short-term postoperative pain, while chronic neuropathic pain studies were included when they informed possible acute-pain applications. Conference abstracts were retained when original data were unavailable elsewhere, with acknowledgment of their preliminary nature.

Exclusion Criteria 

Ineligible sources included pediatric populations, chronic pain without relevance to acute pain, animal studies, case reports without substantive pharmacological content, unvalidated computational models, studies of nonselective sodium-channel blockers without relevant comparative context, and non-English publications. 

The sources covered clinical, pharmacokinetic, pharmacodynamic, mechanistic, regulatory, and emerging evidence summarized in Table 1. Clinical evidence included two phase 2 randomized controlled trials, two phase 3 randomized controlled trials reported together, one phase 3 single-arm study, and a phase 2 trial in diabetic peripheral neuropathy [16,20,21]. FDA prescribing information contributed pharmacokinetic, pharmacodynamic, safety, and dosing data. At the same time, mechanistic evidence came from pharmacological characterization, human dorsal root ganglion electrophysiology, activity-dependent effects, early prescribing data, and targeted protein degradation research [12-14,22-24].

**Table 1:** Characteristics of included studies DRG: dorsal root ganglion, FDA: U.S. Food and Drug Administration, N/A: not applicable, NR: not reported, PD: pharmacodynamics, PK: pharmacokinetics, PROTAC: proteolysis targeting chimera, RCT: randomized controlled trial

Source	Study characteristics	Study purpose
Jones et al., 2023 [9]	Phase 2 RCTs: post-abdominoplasty (n=303); post-bunionectomy (n=274)	PK, PD, clinical efficacy
Stewart et al., 2025 [12]	Electrophysiology - human DRG neurons	PD, mechanism
Osteen et al., 2025 [13]	Pharmacology characterization - in vitro	PD, selectivity
FDA Prescribing Information, 2025 [14]	Regulatory document - product label	PK, PD, safety, dosing
Bertoch et al., 2025 [16]	Phase 3 RCTs: post-abdominoplasty (n=1,118); post-bunionectomy (n=1,073)	Clinical efficacy, safety
McCoun et al., 2025 [20]	Phase 3 single-arm; mixed surgical/non-surgical acute pain (n=256)	Clinical efficacy, safety
Uhelski et al., 2025 [21]	Electrophysiology - human DRG neurons	PD, activity dependence
Freeman et al., 2025 [22]	Phase 2 RCT abstract; diabetic peripheral neuropathy (n=192)	Clinical efficacy
Sideris et al., 2025 [23]	Observational study	Real-world use
Chamessian et al., 2025 [24]	Preclinical study - PROTAC development	Future directions

Suzetrigine Pharmacokinetics 

Pharmacokinetic data were primarily obtained from FDA prescribing information [14]. Oral suzetrigine reaches peak plasma concentration at approximately three hours, while its active metabolite, M6-SUZ, peaks near 10 hours. Food delays absorption without materially altering overall exposure [14]. Suzetrigine has an apparent volume of distribution of 495 L and is 99% protein-bound, compared with 96% for M6-SUZ. Hepatic metabolism occurs mainly through CYP3A, and M6-SUZ retains NaV1.8 activity despite approximately 3.7-fold lower potency than the parent compound [14,25]. Elimination half-lives are approximately 24 hours for suzetrigine and 33 hours for M6-SUZ, supporting twice-daily administration and steady state within three to five days [14]. Strong CYP3A inhibitors substantially increase exposure and are contraindicated; moderate inhibitors require dosage reduction, and strong inducers should be avoided. Suzetrigine also reduces exposure to sensitive CYP3A substrates, including midazolam and potentially hormonal contraceptives [14]. Age, sex, body composition, and mild-to-moderate renal impairment do not require dosage adjustment, whereas moderate hepatic impairment does. Data remain limited in severe hepatic impairment and advanced kidney disease [14]. Pharmacokinetic parameters are summarized in Table 2.

**Table 2:** Pharmacokinetic parameters of suzetrigine AUC: area under the concentration - time curve, CYP3A: cytochrome P450 3A, NR: not reported; Tmax: time to reach maximum concentration, Cmax: highest rate of absorption, eGFR: estimated glomerular filtration rate

Parameter	Suzetrigine	M6-SUZ (active metabolite)	Clinical implication
Time to peak concentration (Tmax)	3 hours	10 hours	Loading dose compensates for delayed steady state
Volume of distribution	495 L	NR	Extensive tissue distribution
Plasma protein binding	99%	96%	High protein binding
Primary metabolic pathway	CYP3A	CYP3A	High drug interaction potential
Relative potency (vs. parent)	1.0 (reference)	↓3.7x	Metabolite contributes meaningfully to activity
Elimination half-life	23.6 hours	33 hours	Supports twice daily dosing
Mean accumulation ratio	3.4	4.5	Steady state achieved in 3-5 days
Excretion route	Feces: 9.1%; urine: primarily as metabolites	Feces: 40.8%; urine 44.0%	Predominantly as metabolites
Effects of food	Delays Tmax (3 h>5 h); Cmax and AUC unchanged; second 50 mg dose unaffected	Delays Tmax (8-10 h>24 h); Cmax and AUC unchanged; second 50 mg dose unaffected	Can be taken with or without food, as food only delays absorption; cannot be taken with grapefruit
Renal impairment (eGFR ≥15 mL/min)	PK unchanged across age, sex, weight, race, and renal function		No dose adjustment required
Hepatic impairment (moderate; Child-Pugh B)	↑AUC 1.5x	↑AUC 1.2x	Dose adjustment required

Suzetrigine Efficacy Outcomes  

Suzetrigine demonstrates high selectivity for NaV1.8, with an IC50 of 3 nM and greater than 30,000-fold selectivity over NaV1.1-NaV1.7 and NaV1.9 [13]. Screening against 180 additional targets showed no significant off-target activity at therapeutic concentrations [13]. Because NaV1.8 is expressed predominantly in peripheral dorsal root and trigeminal ganglion neurons, with minimal expression in the CNS, cardiac tissue, and skeletal muscle, suzetrigine can inhibit peripheral pain signaling while largely sparing central, cardiac, and motor functions [9,11,12]. It binds allosterically to the VSD2 domain and stabilizes NaV1.8 in a closed, nonconducting state [13,15,25]. Its state-dependent and reverse use-dependent properties distinguish it from traditional sodium-channel blockers [25]. In human dorsal root ganglion neurons, suzetrigine attenuates, but does not eliminate, action potential generation or repetitive firing [12]. Residual firing may persist because of large remaining NaV1.8 currents or contribution from NaV1.7, providing a possible explanation for incomplete analgesia and supporting use within multimodal therapy rather than as a complete opioid replacement [9,12]. Activity-dependent findings suggest greater suppression in neurons with abnormal pain signaling, although these findings remain preliminary [21]. Cardiac studies showed no clinically significant QTc prolongation at twice the maximum recommended exposure [13,14].

Phase 2 dose-ranging studies included 303 patients after abdominoplasty and 274 after bunionectomy [9]. High-dose suzetrigine, consisting of a 100-mg loading dose followed by 50-mg maintenance doses, produced greater SPID48 improvement than placebo after both procedures. The least-squares mean differences were 37.8 after abdominoplasty and 36.8 after bunionectomy, while lower doses performed similarly to placebo [9]. These findings established a steep dose-response threshold and informed the phase 3 regimen. Phase 3 trials included 1,118 abdominoplasty patients and 1,073 bunionectomy patients assigned to suzetrigine, hydrocodone/acetaminophen, or placebo [14,16]. Suzetrigine significantly exceeded placebo in both trials, with SPID48 differences of 48.4 after abdominoplasty and 29.3 after bunionectomy [16]. Median time to meaningful pain relief after abdominoplasty was 119 minutes with suzetrigine versus 480 minutes with placebo [14,16]. Suzetrigine produced numerically comparable efficacy to hydrocodone/acetaminophen after abdominoplasty but was less effective than the opioid comparator after bunionectomy based on descriptive comparisons only. These findings suggest that efficacy may vary by pain intensity and mechanism of injury [16]. Clinical efficacy outcomes are summarized in Table 3.

**Table 3:** Summary of clinical trial efficacy outcomes APAP: acetaminophen, CI: confidence interval, HC: hydrocodone, LS: least squares, LSMD: least squares mean difference, NRS: numeric rating scale, SAE: serious adverse event, SPID48: summed pain intensity difference over 48 hours, TEAE: treatment-emergent adverse event

Trial, year, country	Population	N	Primary endpoint	Suzetrigine vs placebo	Suzetrigine vs active comparative
NCT05034952, 2022, USA [9]	Post-abdominoplasty	303	SPID48	Greater pain reduction at high dose (LSMD 37.8; 95% CI 9.2-66.4); lower doses similar to placebo	Not superior to HC/APAP
NCT04977336, 2021, USA [9]	Post-bunionectomy	274	SPID48	Greater pain reduction at high dose only (LSMD 36.8; 95% CI 4.6-69.0)	Not superior to HC/APAP
NCT05558410, 2023, USA [16]	Post-abdominoplasty	1,118	SPID48	Greater pain reduction (LSMD 48.4; 95% CI 33.6-63.1); faster onset (119 vs. 480 min)	Numerically comparable to HC/APAP (LSM: 6.6; 95% CI: -5.4 to 18.7) (descriptive comparison only)
NCT05553366, 2023, USA [16]	Post-bunionectomy	1,073	SPID48	Greater pain reduction (LSMD 29.3; 95% CI 14.0-44.6)	Not superior to HC/APAP
NCT05661734, 2023, USA [20]	Mixed acute pain	256	Patient satisfaction	83.2% rated analgesia good/excellent; TEAEs 36.7%; SAEs 0.78%	No comparator
NCT05660538, 2023, USA [22,26]	Diabetic peripheral neuropathy	192	NRS change at 12 weeks	Pain reduction comparable across all doses (LS mean -2.18 to -2.26)	Numerically comparable to pregabalin (descriptive comparison only); >50% responders higher with low dose (41.7% vs 22.4%)

Suzetrigine Adverse Events 

Across postoperative trials, suzetrigine showed favorable short-term tolerability. Common adverse events included headache, pruritus, constipation, nausea, and dizziness and were generally mild to moderate [9,14,16]. Serious adverse events were rare, and nausea and vomiting occurred less frequently than with hydrocodone/acetaminophen [16]. In a phase 3 single-arm study of 256 patients with surgical and nonsurgical acute pain, 83.2% rated suzetrigine’s effectiveness as good or excellent [20]. Treatment-emergent adverse events occurred in 36.7%, while two serious events were judged unrelated to the medication [20]. In diabetic peripheral neuropathy, suzetrigine produced pain reductions similar to pregabalin across the 23-, 46-, and 69-mg groups [22]. Pregabalin caused more dizziness, peripheral edema, and weight gain, whereas decreased creatinine clearance was observed in some patients receiving 69 mg of suzetrigine [22]. These findings suggest potential benefit across multiple pain modalities, although perioperative and neuropathic pain differ mechanistically. Preliminary observational evidence also indicates early real-world adoption following regulatory approval, but detailed outcomes remain unavailable [23]. Adverse events are summarized in Table 4.

**Table 4:** Summary of adverse events in clinical trials of suzetrigine APAP: acetaminophen, DPN: diabetic peripheral neuropathy, HC: hydrocodone, NR: not reported

Trial/comparator	Nausea	Vomiting	Headache	Dizziness	Constipation	Decreased creatinine renal clearance	Peripheral edema
HC/APAP (phase 3 postoperative) [16]	32.8% (abdominoplasty); 14.4% (bunionectomy)	4.0% (abdominoplasty); 4.4% (bunionectomy)	7.1% (abdominoplasty); 10.4% (bunionectomy)	5.4% (abdominoplasty); 5.3% (bunionectomy)	8.7% (abdominoplasty); 5.1% (bunionectomy)	NR	NR
Suzetrigine (phase 3 postoperative) [16,20]	19.0% (abdominoplasty); 8.2% (bunionectomy)	2.2% (abdominoplasty); 1.6% (bunionectomy)	4.2% (abdominoplasty); 4.9% (bunionectomy)	4.0% (abdominoplasty); 3.5% (bunionectomy)	10.5% (abdominoplasty); 3.5% (bunionectomy)	NR	NR
Suzetrigine (DPN trial) [26]	NR	NR	5.5% (mid dose); 1.8% (high dose)	1.8% (mid dose)	NR	1.8% (mid dose); 10.9% (high dose)	3.6% (low dose)
Pregabalin (DPN trial) [26]	NR	NR	3.70%	9.30%	NR	1.90%	5.60%

Important evidence gaps remain. Postoperative trials have been limited to ambulatory soft-tissue and podiatric procedures, and further research is needed in thoracotomy, laparotomy, joint replacement, and other operations associated with greater pain and opioid exposure [9,16]. Short follow-up periods limit conclusions regarding long-term safety, rare adverse events, and durability of benefit [14]. Exclusion of older patients and those with substantial comorbidities, organ dysfunction, or polypharmacy also limits generalizability [14,16]. No trials have directly compared suzetrigine with optimized multimodal regimens containing nonsteroidal anti-inflammatory drugs (NSAIDs), acetaminophen, gabapentin, or regional anesthesia, and interactions with commonly used perioperative medications remain inadequately studied [3,4]. Recovery-centered outcomes such as mobility, gastrointestinal recovery, length of stay, and return to baseline activity have also not been systematically evaluated [3,4]. Combined NaV1.7 and NaV1.8 inhibition may offer additional benefit, while proteolysis-targeting chimera technology represents a possible future approach to sodium-channel modulation but remains at an early preclinical stage [12,24].

Discussion 

This scoping review identifies suzetrigine as a selective NaV1.8 inhibitor with potential application in perioperative pain management. Available evidence demonstrates predictable oral pharmacokinetics supporting twice-daily dosing, pronounced peripheral target selectivity, and analgesic efficacy exceeding placebo in controlled postoperative trials [9,13,14,16]. Its mechanism differs from opioid receptor agonism, cyclooxygenase inhibition, and central calcium-channel modulation [9,11,15]. Suzetrigine reaches peak plasma concentrations within several hours, while the longer-lasting active metabolite M6-SUZ contributes to sustained exposure. Although it may be administered with or without food, its CYP3A metabolism creates clinically important interaction potential in perioperative settings [14].

Suzetrigine stabilizes NaV1.8 in a nonconducting state, attenuating rather than completely blocking nociceptor activity [13,25]. Its selectivity and the predominantly peripheral expression of NaV1.8 support analgesia with fewer CNS effects than centrally acting medications [9,11-13]. This distinguishes it from opioids, whose widespread receptor activity contributes to respiratory depression, gastrointestinal dysmotility, sedation, and dependence [1,2,6,8]. Phase 3 trials showed approximately 40% less nausea and vomiting with suzetrigine than with hydrocodone/acetaminophen [14,16].

Its distinct mechanism makes suzetrigine a potential component of multimodal analgesia, although it has not yet been formally evaluated within optimized perioperative regimens [3,4]. Mechanistic studies indicate that residual NaV1.8 current and continued NaV1.7 activity may sustain action-potential firing despite treatment [12]. This explains the incomplete analgesia observed clinically and supports using suzetrigine as part of an analgesic regimen rather than as a complete opioid replacement. The ceiling effect also suggests that doses above the approved regimen are unlikely to produce substantial additional benefit, consistent with phase 2 findings [9]. State-dependent binding and reverse use-dependence may also influence efficacy across different pain states, while preliminary data suggest greater suppression during sustained or abnormal nociceptor activity [21,25].

Current evidence suggests that suzetrigine may be most useful for patients with moderate-to-severe pain who can receive oral therapy, particularly those at increased risk for opioid-related gastrointestinal or respiratory effects [14,16]. The approved regimen is a 100-mg loading dose followed by 50 mg every 12 hours [9,14]. Dose reduction is required with moderate CYP3A inhibitors and moderate hepatic impairment; strong CYP3A inhibitors are contraindicated, and strong inducers should be avoided [14]. Preliminary prescribing evidence suggests early clinical adoption, although its optimal place within perioperative protocols remains uncertain [23].

Important evidence gaps remain because postoperative trials have largely involved ambulatory soft-tissue and foot procedures [3]. Studies are needed in major operations and should assess opioid consumption, recovery, patient-reported outcomes, healthcare utilization, cost, and adverse events. Larger real-world studies are also required in patients with comorbidities, organ dysfunction, polypharmacy, or prolonged exposure [23]. Combined NaV1.7 and NaV1.8 inhibition may offer additional benefit, while targeted protein degradation represents a possible future approach [12,24]. This review is also limited by the absence of prospective registration, the English-language restriction, inclusion of conference abstracts and preprints, limited independent replication, and the lack of a formal risk-of-bias assessment [21-24]. Suzetrigine therefore represents a promising non-opioid option, but broader evidence, clinician education, cost evaluation, and postmarketing surveillance remain necessary before widespread perioperative integration.

## Conclusions

This scoping review analyzes the pharmacokinetic, pharmacodynamic, and clinical evidence supporting the use of suzetrigine as the first selective NaV1.8 inhibitor approved for acute pain management. The compiled literature describes predictable oral pharmacokinetics supporting twice-daily dosing, exceptionally high selectivity over other sodium-channel isoforms, and reproducible analgesic efficacy across phase 2 and phase 3 postoperative trials. Mechanistic accounts in the literature indicate that NaV1.8 inhibition diminishes but does not eliminate nociceptor activation, providing a biological explanation for meaningful but incomplete clinical analgesia. Its state-dependent binding and reverse use-dependence observed in these studies distinguish it from traditional sodium-channel blockers and support its use as part of multimodal analgesia rather than as a standalone opioid replacement.

Critical gaps remain regarding major surgical populations, comparative effectiveness against optimized multimodal regimens, real-world use, and long-term safety. Preliminary evidence in neuropathic pain and emerging approaches such as targeted protein degradation suggests broader future applications, but systematic evaluation remains necessary. Overall, the literature demonstrates that peripheral NaV1.8 blockade represents a pharmacologically innovative non-opioid strategy warranting continued investigation and thoughtful integration into contemporary perioperative pain management. Further larger studies and systematic reviews are required to support these findings.
